# Association of serum uric acid with hypertriglyceridemia in children and adolescents: a cross-sectional study

**DOI:** 10.1186/s12944-024-02182-1

**Published:** 2024-06-24

**Authors:** Shang-An Si, Meng-Qi Chen, Gui-Ju Zhang

**Affiliations:** 1 The First Clinical Medical College, Shandong University of Traditional Chinese Medicine, Shandong, 250014 China; 2 Affiliated Hospital of Shandong University of Traditional Chinese Medicine, Shandong, 250014 China

**Keywords:** Serum uric acid, Hypertriglyceridemia, NHANES, Children, Adolescent

## Abstract

**Background:**

Uric acid (UA), a liver-derived metabolite, is intimately tied to metabolic disorders. Although ample research underscores its connection with hypertriglyceridemia (HTG), studies focusing on adolescents remain limited. To fill the gaps in epidemiology,this study focused on analyzing the relationship between the levels of uric acid and HTG in a demographic sample comprising adolescents from the United States.

**Methods:**

In this study, a total of 4,435 participants through the National Health and Nutrition Examination Survey (NHANES) from 2011 to 2020. The exposure variable was serum uric acid (SUA), the effect variable was HTG, and the covariates included demographic, questionnaire, physical examination and laboratory indicators. We utilized weighted logistic regression and meticulous subgroup evaluations to discern the intrinsic link between SUA and HTG. Stratified analyses augmented the validation of this association, while smooth curve fitting probed for potential nonlinear correlations.

**Results:**

The study included 4,435 participants. Male adolescents exhibit elevated SUA levels. After adjusting for all variables, the weighted multiple logistic regression model revealed that SUA was positively correlated with HTG risk (OR = 1.006, 95% CI: 1.005–1.007). This relationship was consistent across the three tertiles group of SUA (T1: OR = 1.006 [95% CI: 1.005–1.007]; T2: OR = 1.006 [95% CI: 1.005–1.007]; T3: OR = 1.004 [95% CI: 1.003–1.006]; P for trend < 0.001). Stratified analyses confirmed that the positive correlation between SUA and HTG risk was significant, irrespective of sex, age or race.

**Conclusions:**

In American children and adolescents aged 12 to 18 years, there was a pronounced association between SUA and HTG. SUA could serve as a risk indicator for HTG. It is recommended that children diagnosed with HTG should be regularly tested for SUA levels. In addition, it is recommended that SUA be included in the comprehensive care of children diagnosed with HTG.

**Supplementary Information:**

The online version contains supplementary material available at 10.1186/s12944-024-02182-1.

## Introduction

Within the United States (US), the prevalence of hypertriglyceridemia (HTG) is 10.7% among youths aged 12 to 19 years [1]. Pediatric dyslipidemia can increase susceptibility to cardiovascular afflictions later in life—a predominant mortality factor in the nation [2]. Furthermore, HTG not only heightens cardiovascular threats but also exhibits a profound correlation with obesity, diabetes, and nonalcoholic steatohepatitis [3–5]. Consequently, vigilant surveillance of lipidemia in young people, discerning dyslipidemia precursors, and preemptively mitigating the inception and advancement of HTG has become paramount.

Serum uric acid (SUA), an end-product of purine catabolism, is linked to noncommunicable ailments, including hypertension, coronary arteriopathy, and metabolic syndrome [6–8]. Concurrently, augmented SUA concentrations resonate with an escalated peril of both holistic and cardiovascular demise [9, 10]. In a study of Chinese children and adolescents, SUA levels increased significantly between the ages of 11 and 15 years, with an estimated prevalence of hyperuricemia in children reaching 23.3% [11]. As a pro-oxidant, SUA contributes to pathological responses in humans by amplifying the oxidative stress response, which is typically viewed as one of the primary mechanisms underlying metabolic and cardiovascular diseases [4].

A growing number of studies have shown a correlation between SUA and HTG. Related research has shown that high levels of UA induce mitochondrial oxidative stress and promote the progression of HTG and metabolic syndrome [12, 13]. Other studies have shown that the apolipoprotein e (ApoE) allele is associated with increased SUA levels [14]. SUA affects residue receptors and reduces the level of very low-density lipoprotein (VLDL) in the liver, eventually leading to elevated triglyceride levels [15]. An increasing number of studies have shown that UA can be considered a risk factor for HTG, and it is suggested that monitoring and controlling SUA levels can prevent and treat HTG and its complications [16–18]. In a clinical study of the effect of uric acid-lowering therapy on HTG in patients with gout, serum triglycerides were reduced with the use of uric acid-lowering agents such as febuxostat and benzbromarone [16]. A longitudinal population-based epidemiologic study revealed that SUA predicts the occurrence of HTG in a positive and dose-dependent manner. Mechanistically, high UA may inhibit enzymes that catalyze the breakdown of triglycerides and reduce the breakdown of serum triglycerides, leading to a greater incidence of HTG in people with high UA than in healthy people [19]. A number of previous studies have shown that there is a correlation between SUA and various components of metabolic syndrome in children, among which triglycerides and high-density lipoprotein have shown a more significant correlation with UA [20–22].

However, the association between SUA and HTG remains ambiguous, particularly in children and adolescents. Consequently, data from the National Health and Nutrition Examination Survey (NHANES) database (2011–2020) were utilized in a cross-sectional study to provide epidemiological evidence.

### Methods

#### Data sources

The data were obtained from the NHANES repository. The National Health Survey (NHANES), a biannual national cross-sectional survey, aims to collect insights into the health and nutritional landscape of American households. The interview segment encompasses demographic, socioeconomic, and dietary nutrition inquiries. The laboratory test segment comprises various examinations [23]. The study protocols were all approved by the ethical review board of the National Center for Health Statistics, and written consent was obtained from all participants before data collection. Relevant published study data are available at https://www.cdc.gov/nchs/nhanes/. This study rigorously adhered to the Strengthening Reporting of Observational Studies in Epidemiology (STROBE) guidelines for cross-sectional studies [24].

#### Study population

**Fig. 1:**
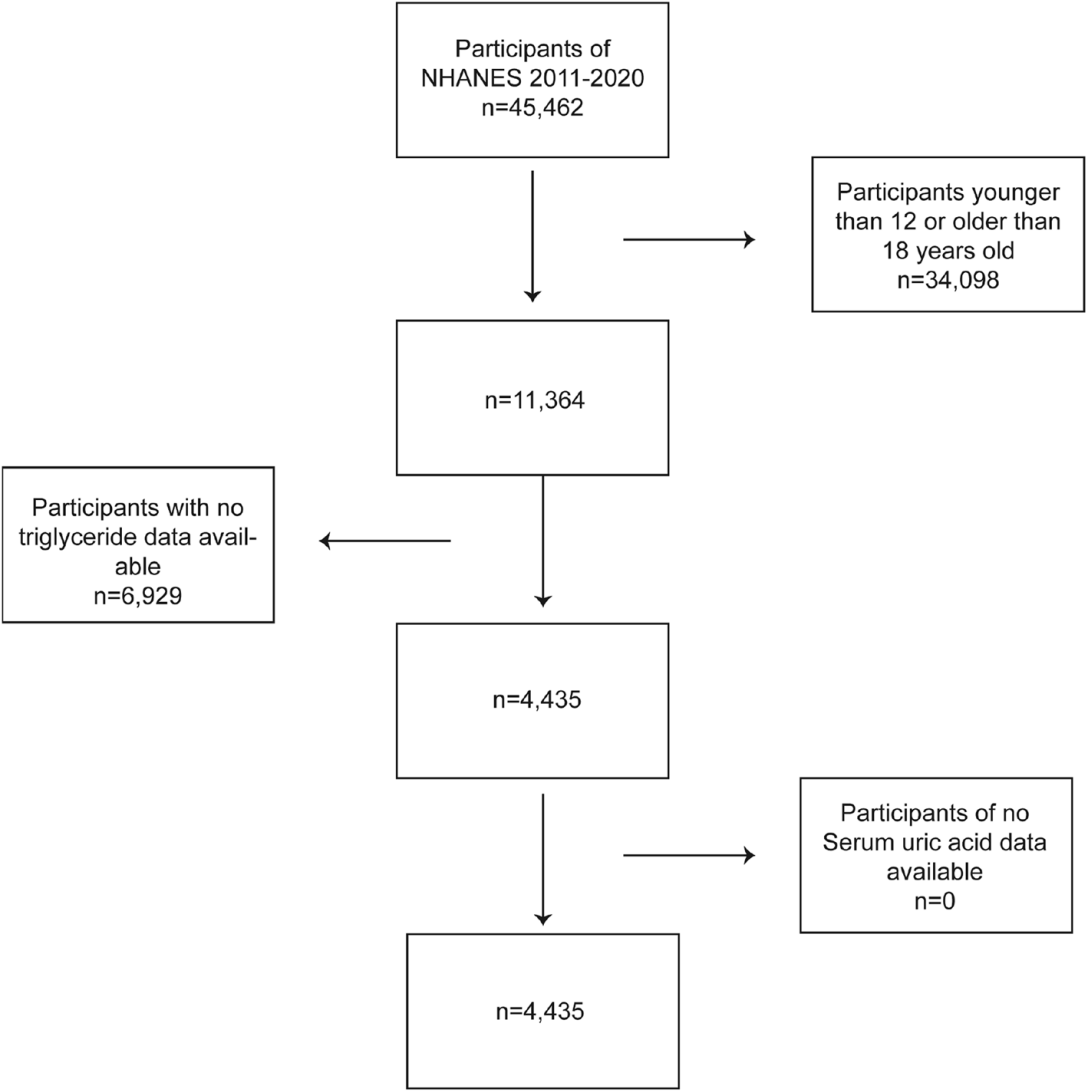
Flowchart of the sample selection for this study

The analysis utilized data from four NHANES cycles spanning the years 2011 to 2020. The dataset consisted of a total of 45,462 observations, encompassing 11,364 adolescents aged between 12 and 18 years who underwent screening. The subsequent exclusion of 6,929 children and adolescents with incomplete data on serum triglyceride and SUA levels resulted in a final sample size of 4,435 participants (Fig. 1).

#### Definition of HTG

This cross-sectional investigation focused on HTG as the principal outcome. HTG was defined as a serum triglyceride level greater than 150 mg/dL (> 17 mmol/L) [25, 26]. The Collaborative Studies Clinical Laboratory at the University of Minnesota Medical Center gauged these levels utilizing the Roche/Hitachi Modular P Chemistry Analyzer. Further pertinent standards are available at http://cdc.gov/nchs/nhanes.

#### Measurement of SUA

This cross-sectional investigation focused on SUA as the main dependent variable. SUA was determined by the production of colored products from the oxidation of UA through the action of 4-aminophenazone (fluorescence method). The results are reported with one decimal place. Low reported results (< 0.2 mg/dL) were reported. Details are provided on the NHANES website: http://cdc.gov/nchs/nhanes.

#### Other covariates

Based on prior research concerning factors correlated with HTG in the youthful demographic and cognizant of the pronounced disparities in individual ramifications from potential confounders [27–30], we incorporated the following variables for comprehensive adjustment: continuous variables: Albumin (g/dL), Alanine Aminotransferase (ALT, u/L), Aspartate Aminotransferase (AST, u/L), Alkaline phosphatase (ALP, U/L), Creatinine (umol/L), Gamma glutamyl transferase (GGT, U/L), Glucose (mmol/L), Lactate dehydrogenase (LDH, U/L), Total protein (g/dL), systolic blood pressure (SBP, mmHg), diastolic blood pressure (DBP mmHg), and dietary data [Total saturated fatty acids (g), Total monounsaturated fatty acids (g) and Total polyunsaturated fatty acids (g)] and categorical variables: sex (male, female) and race (non-Hispanic white, non-Hispanic Black, Mexican American and other).

#### Statistical analysis

The NHANES weights were incorporated into all calculations to more accurately capture the comprehensive nuances of the data. The purpose of applying weights is to adjust the coefficients of each independent variable in order to reflect the varying degrees of influence that different independent variables have on the dependent variable, thereby enabling research findings to better represent the overall population of the United States [23, 31]. The analytical process was conducted in four stages. Initially, SUA concentrations were divided into three groups based on tertile distinctions. Chi-square assessments were employed for categorical distinctions, while weighted t-tests were used for continuous variables. Subsequently, a weighted multifactorial logistic regression analysis elucidated the discrete correlation between SUA and HTG, controlling for various variables. The initial modulation accounted for sex, age, and ethnicity, while the subsequent one incorporated all covariates. The advantage of utilizing a multivariate logistic regression model lies in its ability to consider the influence of multiple factors on the outcome, thereby yielding more precise prediction results. Moreover, such analytical frameworks are instrumental in assessing the impact of various factors on outcomes, thereby enhancing our comprehension of the dataset. In the third step, a stratified analysis scrutinized the influence of disparate subgroups on the outcomes. In addition, the linear relationship between SUA and HTG was elucidated by smooth curve fitting. The significance threshold was set at *P* < 0.001. All analysis was performed using R software, version 3.4.3, and EmpowerStats Software (www. empowerstats.com).

## Results

### Baseline characteristics of the participants

Participants were categorized into three distinct groups based on their SUA tertile rankings. Table 1 provides a comprehensive overview of the fundamental characteristics of the entire cohort consisting of 4,435 subjects, organized according to these SUA tertiles. The characteristics exhibited significant variations across SUA tertiles. In comparison to other participants, males constituted the majority (79.9%) in the highest SUA tertile. There were notable disparities observed between SUA tertiles and ALT, AST, creatinine, GGT, LDH, total protein levels, blood pressure readings, and dietary TMFA intake (*P* < 0.001).

**Table 1:** Characteristics of serum uric acid levels in the study population

Characteristics	Serum Uric acid(umol/L)			*P* value
	T1 (< 225.8)	T2 (225.8–327.1)	T3 (> 327.1)	
Albumin (g/dL)	4.338 ± 0.327	4.410 ± 0.313	4.478 ± 0.315	< 0.001
ALT (U/L)	15.078 ± 7.909	16.640 ± 8.768	22.356 ± 16.871	< 0.001
AST (U/L)	20.993 ± 8.520	21.862 ± 6.983	24.191 ± 10.113	< 0.001
ALP (U/L)	130.839 ± 90.466	151.051 ± 99.319	149.468 ± 92.671	< 0.001
Creatinine (umol/L)	55.448 ± 11.523	61.508 ± 13.623	69.667 ± 14.930	< 0.001
GGT (U/L)	12.508 ± 7.865	13.774 ± 9.168	17.824 ± 11.073	< 0.001
Glucose (mmol/L)	4.905 ± 0.721	4.917 ± 0.720	4.993 ± 0.846	0.003
LDH (U/L)	138.398 ± 35.557	140.841 ± 32.589	144.449 ± 35.840	< 0.001
Total protein (g/dL)	7.197 ± 0.412	7.238 ± 0.412	7.329 ± 0.414	< 0.001
Dietary intake				
TSFA (g)	26.063 ± 15.733	27.525 ± 17.197	27.914 ± 18.021	0.010
TMFA (g)	25.599 ± 14.825	27.497 ± 16.637	28.524 ± 17.806	< 0.001
TPFA (g)	17.897 ± 11.269	18.779 ± 12.628	19.464 ± 13.089	0.004
SBP (mmHg)	106.772 ± 7.788	108.210 ± 8.067	111.455 ± 9.014	< 0.001
DBP (mmHg)	60.286 ± 10.210	60.474 ± 9.873	61.581 ± 10.417	< 0.001
Sex (%)				< 0.001
Male	23.2	49.4	79.9	
Female	76.8	50.6	20.1	
Age (years)	14.790 ± 2.040	14.826 ± 1.943	15.311 ± 1.907	< 0.001
Race				< 0.001
Mexican American	22.1	19.5	19.1	
Non-Hispanic White	23.5	28.0	29.9	
Non-Hispanic Black	28.3	25.0	19.5	
Other Race/ethnicity	26.1	27.5	31.5	
Hypertriglyceridemia(%)				< 0.001
Non-Hypertriglyceridemia	91.1	88.6	80.3	
Hypertriglyceridemia	8.9	11.4	19.7	

### Associations between SUA levels and HTG

**Fig. 2:**
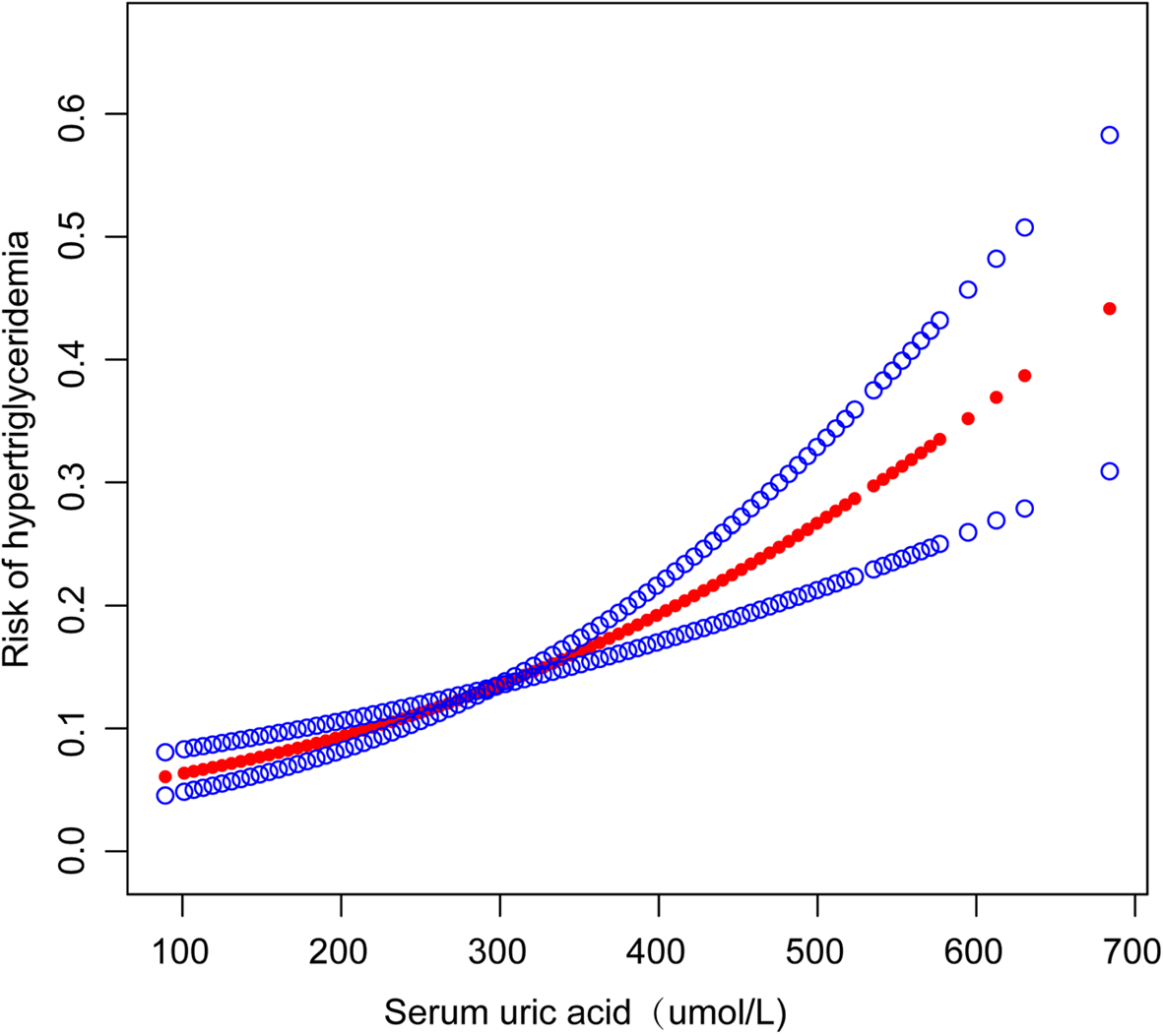
The correlation between serum uric acid and hypertriglyceridemia. (Red lines indicate smooth fit curves between variables. The blue lines indicate the 95% confidence intervals of the fits. Age, sex, race, glucose, albumin, ALT, AST, LDH, ALP, total protein, GGT, creatinine, SBP, DBP, TMFA, TPFA and TSFA were adjusted

The outcomes of the multifactorial regression assessment are presented in Table 2. Model 1 demonstrates a significant positive association between SUA and the onset of HTG, without considering other factors (OR = 1.006, 95% CI: 1.005–1.007, *P* < 0.001). The adjusted Model 2, accounting for age, gender, and race, revealed a statistically significant positive association (OR = 1.006, 95% CI: 1.005–1.007, *P* < 0.001). Moreover, even after comprehensive adjustment for all covariates in Model 3, the levels of SUA still demonstrated a positive correlation with HTG (OR = 1.004, 95% CI: 1.003–1.006, *P* < 0.001). The relationship between SUA and HTG was comprehensively examined by dividing SUA into tertiles. In the fully adjusted model 3, when comparing the highest tertile (T3) with the lowest tertile (T1), an odds ratio (OR) of 1.941 (95% CI: 1.461–2.579) was obtained, indicating a consistent positive correlation between elevated levels of SUA and HTG. Their interrelation underwent scrutiny via a generalized additive framework and polished curve adaptation (penal spline methodology), yielding congruent revelations (Fig. 2).

**Table 2:** Analysis of Hypertriglyceridemia risk in adolescents aged 12–18 years based on data from NHANES 2011–2020, including 4,435 participants

Serum Uric acid(umol/L)	Hypertriglyceridemia OR (95% CI) *P* value		
	Model 1	Model 2	Model 3
Per 1SD increase	1.006 (1.005, 1.007) < 0.001	1.006(1.005, 1.007) < 0.001	1.004 (1.003, 1.006) < 0.001
Serum Uric acid (tertiles)			
T1 (< 225.8)	1.0	1.0	1.0
T2 (225.8–327.1)	1.320 (1.035, 1.683) 0.0251	1.302(1.013, 1.674) 0.0392	1.294 (1.000, 1.674) 0.0499
T3 (> 327.1)	2.507 (1.997, 3.148) < 0.001	2.401(1.851, 3.115) < 0.001	1.941 (1.461, 2.579) < 0.001
*P* for trend	< 0.001	< 0.001	< 0.001

Model 1 was no confounder

Model 2 was adjusted for age, sex, and race

Model 3 was adjusted for age, sex, race, glucose, albumin, ALT, AST, LDH, ALP, total protein, GGT, creatinine, SBP, DBP, TMFA, TPFA, and TSFA

### Subgroup analyses

**Fig. 3:**
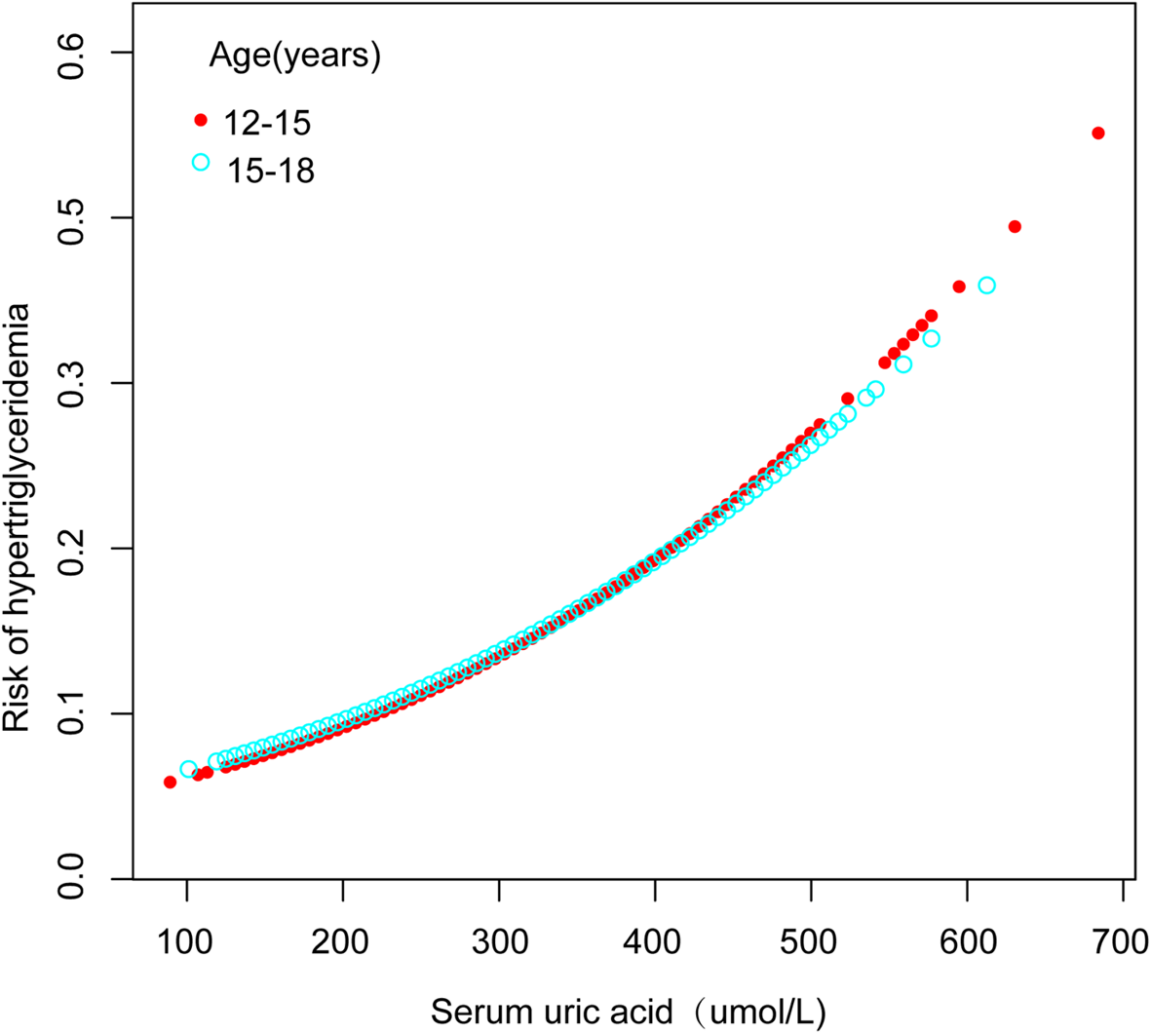
The correlation between serum uric acid and hypertriglyceridemia stratified by age

**Fig. 4:**
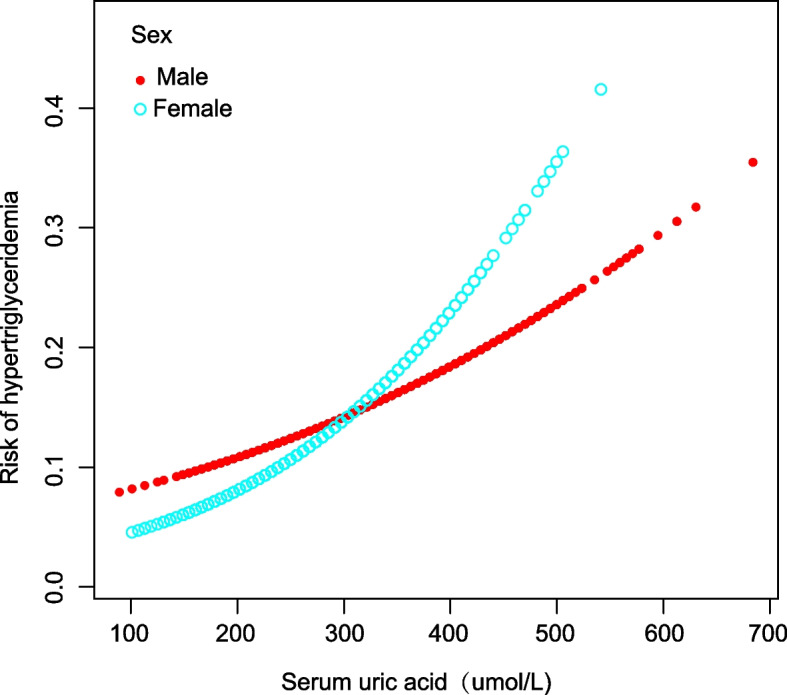
The correlation between serum uric acid and hypertriglyceridemia stratified by sex

**Fig. 5:**
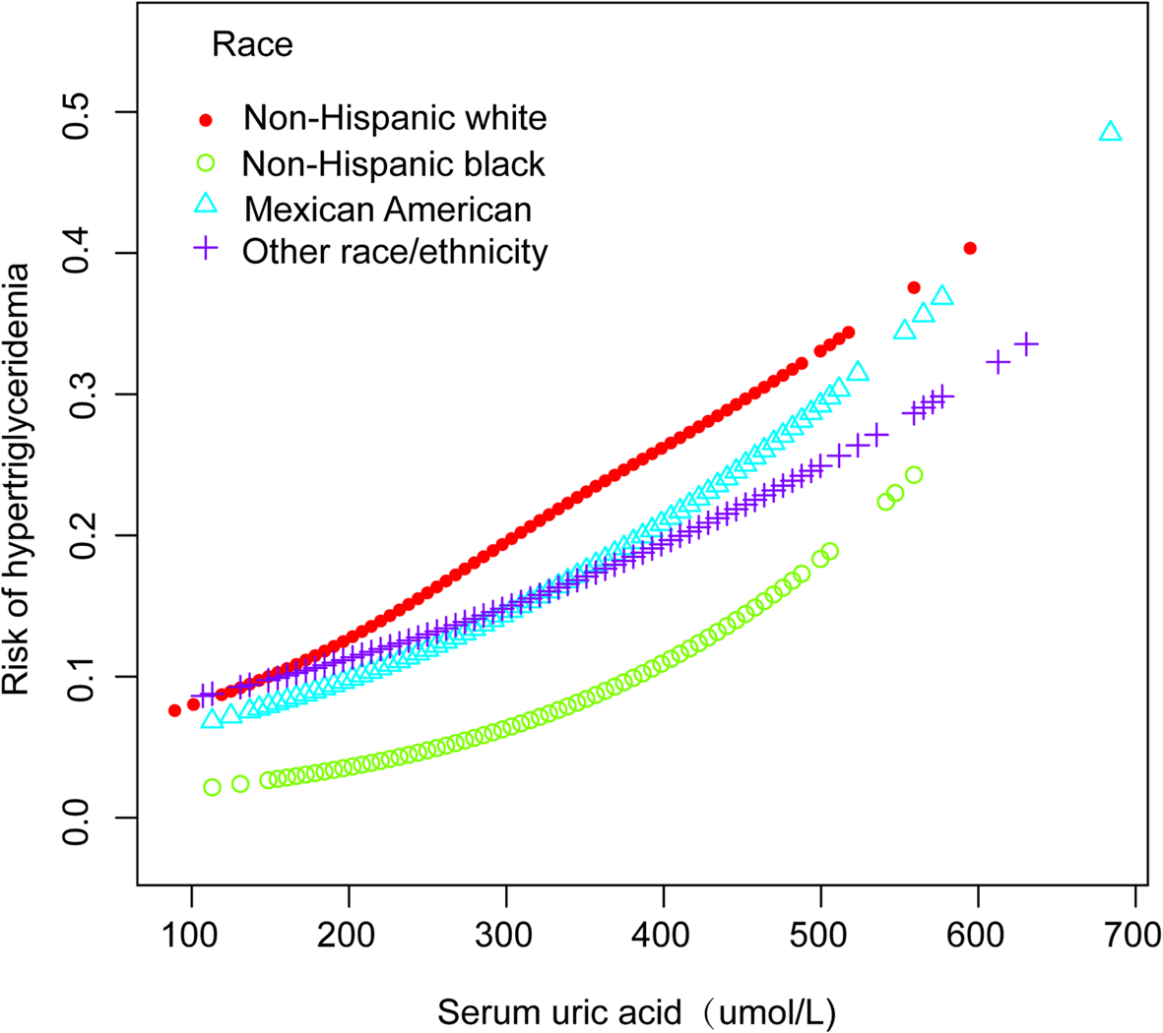
The correlation between serum uric acid and hypertriglyceridemia stratified by race

Further analysis within subgroups, delineated by factors such as age, sex, and ethnicity, consistently revealed a significant correlation between SUA concentrations and the incidence of HTG, as evidenced in Table 3. In addition, the nonlinear association between the two parameters was assessed through smooth curve fitting, which yielded consistent outcomes (Figs. 3, 4, and 5). In addition, from the smooth curve fitting plot, we hypothesize that the risk of HTG is lowest in Non-Hispanic Black and highest in Non-Hispanic White at the same SUA level.

**Table 3:** Subgroup analyses of the effect of serum uric acid on hypertriglyceridemia

	N	OR (95% CI)	*P* for trend
Sex			
Male	2,292	1.005 (1.004, 1.007)	< 0.001
Female	2,143	1.007 (1.005, 1.010)	< 0.001
Age (years)			
12–15	2,541	1.006 (1.004, 1.007)	< 0.001
15–18	1,894	1.006 (1.004, 1.007)	< 0.001
Race			
Mexican American	893	1.006 (1.004, 1.008)	< 0.001
Non-Hispanic White	1,071	1.008 (1.004, 1.011)	< 0.001
Non-Hispanic Black	1,210	1.006 (1.004, 1.008)	< 0.001
Other Race/ethnicity	1,261	1.004 (1.002, 1.006)	< 0.001

## Discussion

In this study, a higher concentration of SUA was found to be positively associated with an increased prevalence of HTG among adolescents. Subgroup analysis further demonstrated a consistent association between the incidence of HTG in both children and adolescents.

The findings of this study indicated a higher prevalence of elevated levels of UA in men compared to women, which is consistent with previous research [32, 33]. It has been suggested that this disparity may be attributed to the excretory effects of estrogen on UA in the kidneys and the reabsorption effects of androgens on UA in the renal tubules [34, 35]. Additionally, adolescent males are more prone to adopting unhealthy lifestyles and behavioral habits during adolescence, such as smoking, alcohol consumption, and high-fat diets; all of which are potential risk factors for increased serum uric acid levels [36].

To date, numerous studies have investigated the association between SUA and HTG, yet limited attention has been given to the adolescent and pediatric population. A meta-analysis conducted by Goli P et al. demonstrated a robust correlation between SUA concentration and key components of metabolic syndrome in children, including HTG [37]. Consistent with these findings, a previous study in a Chinese community revealed an escalating prevalence of HTG with increasing basal levels of SUA [38]. Civantos Modino S [39] reported that overweight and obese children exhibited elevated average SUA levels, which significantly increased their likelihood of developing metabolic syndrome. Notably, SUA serves as a superior marker for predicting fatty liver risk in adolescents compared to metabolic syndrome itself [40]. In a retrospective analysis of 4,922 children diagnosed with adenoid or tonsil hypertrophy, a one standard deviation increase in serum uric acid (SUA) was associated with a 27% increased risk of dyslipidemia, with an OR of 1.270 and a 95% CI ranging from 1.185 to 1.361. Correspondingly, the incidence of hypertriglyceridemia (HTG) increased, indicating a significant positive correlation between SUA and HTG [41]. Furthermore, a cross-sectional study investigating the relationship between HTG and SUA in adults revealed a positive correlation in the U.S. adult cohort [42], with the association being more pronounced in individuals younger than 60 years, suggesting that the impact of SUA on health is more significant in younger individuals [43].

However, there is no definitive conclusion regarding the mechanism of SUA and HTG. Studies have shown that high UA is closely related to metabolic syndrome, a view further confirmed in the adolescent population [44]. The potential mechanism of action between the two includes the excessive production of reactive oxygen species by UA through the enzymatic action of xanthine oxidase, which causes endothelial dysfunction [45]. Concurrently, when soluble UA impairs nitric oxide production in endothelial cells, it also induces endothelial dysfunction [46]. These findings were confirmed in a population of obese prepubertal children [47]. Previous studies have confirmed that UA enters cells through organic ionophores and induces strong oxidation [48]. This significantly increases the burden of liver metabolism and fat accumulation. As mentioned earlier, oxidative stress responses within the vast majority of cells activate sterol regulatory element-binding proteins, resulting in the production of various lipogenic enzymes [49]. All these lipases promote the production of fat, especially in liver cells, which greatly increases the risk of nonalcoholic fatty liver disease [50]. Moreover, UA induces protein kinase B phosphorylation by mediating mitochondrial oxidative stress, affecting endothelial function [51, 52]. In an animal experiment, UA was observed to upregulate the expression of miR-149-5p and regulate the miR-149-5p/FGF21 axis, resulting in triglyceride accumulation in the liver [53]. Meng et al. [54] first proposed that the CXCL-13 pathway is a key factor that is affected by hyperuricemia and disrupts lipid metabolic processes in vivo and in vitro. Moreover, prevailing conjectures posit that augmented SUA concentrations could attenuate lipase efficacy, thus curbing the catabolism of triglycerides [55]. Contemporary research delineates a salient affirmative association between SUA and triglyceride-abundant lipoprotein cholesterol (TRL-C). This association might be mediated by bridging effects, such as the high expression of angiopoietin-like protein 4 (ANGPTL4) in subcutaneous adipose tissue and the inhibition of lipoprotein lipase activity [56]. These findings indicate that UA, which plays a direct or indirect role in cellular oxidative stress, is implicated in lipid metabolism disorders.

### Study strengths and limitations

A significant advantage of this investigation lies in its expansive sample size, comprising 4,435 participants. This extensive collection of data bolsters the study's capacity to delineate a precise correlation between SUA concentrations and the incidence of HTG among children and adolescents. By encompassing a broad demographic, this research provides robust evidence that underscores the relationship between SUA levels and HTG, offering a clearer understanding of metabolic health in the younger population. Second, based on clinical experience and prior data, relevant covariates were chosen to mitigate study bias. Finally, the data are sourced from the NHANES database, which can better represent the population characteristics of the native US after quoting sampling weights.

However, some limitations exist. For example, the included data represent only the U.S. population. Therefore, caution should be exercised when extrapolating findings to other countries or races. Furthermore, the study results may be influenced by unmeasured variables. Additionally, it is important to note that the cross-sectional design of this study limits our ability to establish a causal relationship between UA and hyperlipidemia. These findings suggest that future research should focus on elucidating the underlying mechanisms linking SUA and HTG in order to validate our conclusions.

## Conclusions

In conclusion, this investigation has revealed a compelling correlation between SUA concentrations and HTG, indicating that elevated SUA levels contribute to the development of HTG in American youth. Importantly, these findings underscore the significance of effectively managing and controlling SUA levels in children and adolescents with HTG to prevent disease progression. Future research should further explore the underlying mechanisms of this association and validate our assertions.

### Supplementary Information

Supplementary Material 1.

## Data Availability

The datasets presented in this study can be found in the following online repositories: https://www.cdc.gov/nchs/nhanes/.
